# Synergy of Pd atoms and oxygen vacancies on In_2_O_3_ for methane conversion under visible light

**DOI:** 10.1038/s41467-022-30434-0

**Published:** 2022-05-25

**Authors:** Lei Luo, Lei Fu, Huifen Liu, Youxun Xu, Jialiang Xing, Chun-Ran Chang, Dong-Yuan Yang, Junwang Tang

**Affiliations:** 1 Key Lab of Synthetic and Natural Functional Molecule Chemistry of Ministry of Education, The Energy and Catalysis Hub, College of Chemistry and Materials Science, Northwest University, Xi’an, PR China; 2 Department of Chemical Engineering, University College London, Torrington Place, London, WC1E 7JE UK; 3 Shaanxi Key Laboratory of Energy Chemical Process Intensification, School of Chemical Engineering and Technology, Xi’an Jiaotong University, Xi’an, PR China; 4 Shaanxi Yanchang Petroleum (Group) Corp. Ltd., Xi’an, 710069 PR China

**Keywords:** Solar energy, Photocatalysis, Catalytic mechanisms

## Abstract

Methane (CH_4_) oxidation to high value chemicals under mild conditions through photocatalysis is a sustainable and appealing pathway, nevertheless confronting the critical issues regarding both conversion and selectivity. Herein, under visible irradiation (420 nm), the synergy of palladium (Pd) atom cocatalyst and oxygen vacancies (OVs) on In_2_O_3_ nanorods enables superior photocatalytic CH_4_ activation by O_2_. The optimized catalyst reaches ca. 100 μmol h^−1^ of C1 oxygenates, with a selectivity of primary products (CH_3_OH and CH_3_OOH) up to 82.5%. Mechanism investigation elucidates that such superior photocatalysis is induced by the dedicated function of Pd single atoms and oxygen vacancies on boosting hole and electron transfer, respectively. O_2_ is proven to be the only oxygen source for CH_3_OH production, while H_2_O acts as the promoter for efficient CH_4_ activation through ·OH production and facilitates product desorption as indicated by DFT modeling. This work thus provides new understandings on simultaneous regulation of both activity and selectivity by the synergy of single atom cocatalysts and oxygen vacancies.

## Abstract (web summary)

CH4 oxidation to high value chemicals under mild conditions through photocatalysis confronts critical issues regarding both conversion and selectivity. Here, atomic Pd and oxygen vacancies were integrated on In2O3 nanorods, leading to visible-driven CH4 conversion with a remarkable yield of oxygenates and high selectivity of primary products.

## Introduction

As the predominant constituent of natural gas, methane hydrate and shale gas resources, selective methane (CH_4_) oxidation to value-added chemicals holds considerable financial and environmental prospective^1–5^. However, the inert symmetrical tetrahedral structure of CH_4_ makes it rather difficult for the dissociation of the first C–H bond, which is the most important step for the activation of methane^6–8^. Industrial multistep route via steam reforming and subsequent Fischer–Tropsch synthesis could efficiently activate CH_4_, while it requires harsh experimental conditions (e.g., >700 °C temperature and/or high pressure), causing huge energy consumption and safety issues^9–13^. In parallel, it is relatively challenging to achieve high selectivity due to the more reactive characteristics of the desired oxygenates against both the reactant CH_4_ and stable product CO_2_^14–17^. Therefore, selective CH_4_ conversion to value-added chemicals under mild conditions other than CO_2_ is highly attractive, while facing considerable challenges.

Photocatalysis offers an appealing alternative to drive many tough redox reactions under mild conditions including CO_2_ conversion^18,19^, N_2_ reduction^20^ and selective CH_4_ oxidation^8^. Recently, various value-added chemicals such as methanol^1,21–24^, formaldehyde^25,26^, ethanol^27,28^, ethane and ethylene^29–34^ were produced by photocatalysis. For example, we found that up to 90% selectivity with a yield of 3.5 μmol h^−1^ methanol could be achieved over the optimized FeO_x_/TiO_2_ photocatalyst under ambient condition using H_2_O_2_ as an oxidant^22^. Recently a high yield of liquid oxygenates including CH_3_OH, CH_3_OOH and HCHO were produced under full arc irradiation over Au supported ZnO, together with the good selectivity of primary products (CH_3_OH and CH_3_OOH) (<70%)^1^. Very recent, the yields of 18.7 μmol h^−1^ HCHO and 3.7 μmol h^−1^ CH_3_OH were reported on quantum BiVO_4_ with an excellent selectivity toward HCHO (87%) and CH_3_OH (99%) under 300–400 nm or 400–780 nm irradiation^26^. Given these significant advances in photocatalytic methane conversion, the yield and/or selectivity to high value chemicals are still quite moderate, in particular it is very challenging to achieve methane activation under visible light irradiation instead of a full arc spectrum due to a narrowed bandgap with mitigated reduction or oxidation potentials.

To realize visible-driven methane oxidation by O_2_ gas on narrow bandgap photocatalysts, the cocatalyst is crucial that not only promotes charge separation, but more importantly manipulates the activation energy of the methane conversion and the selectivity^35–40^. Furthermore rationally regulating the production of reactive oxygen species (ROS) through cocatalyst modification is necessary as ·OH radicals have been widely regarded as the main species that induced CH_4_ activation and overoxidation^41,42^. When CH_3_OH served as the desired products, overoxidation to HCHO or CO_2_ would be suppressed by lowering the oxidative potential of photogenerated hole through cocatalyst modification, thus improving the selectivity. Stimulated by molecular catalysis, single atom cocatalysts promise an extremely high efficiency, where atomic dispersed species with unsaturated coordination environment could improve the catalytic performances based on the unique electronic structure^43–45^. Meanwhile, high atom utilization efficiency could be achieved^46,47^. On the other hand, since CH_4_ exhibited low electron and proton affinity, moderate decoration of defective sites could enhance the chemical-adsorption of non-polar molecular, then promoting the activation of CH_4_^48^. Therefore, the integration of both defects and single atom cocatalyst decoration could boost charge separation, weaken oxidative potential and enhance CH_4_ activation on a photocatalyst.

Herein, atomically dispersed palladium (Pd) supported on defective In_2_O_3_ was prepared and served as the visible-light responsive photocatalyst for CH_4_ conversion to high value chemicals. Under 420 nm irradiation, the production of oxygenates on the photocatalyst reached up to ca. 300 μmol in 3 h, with a very high selectivity of 82.5% over primary products. In situ XPS and EPR spectra were conducted to investigate the charge transfer dynamics. The results indicated the dedicated roles of Pd atoms and oxygen vacancies (OVs) in promoting the transfer of photo-induced holes and electrons, respectively. DFT calculation results indicated H_2_O could also promoted the desorption of the oxygenate products, thus suppressing overoxidation and facilitating high selectivity of primary products. The introduction of both atomic Pd and OVs further enhanced this effect on suppressing overoxidation. Isotopic labeled experiments further proved the methane conversion pathway.

## Results and discussion

### Visible-light photocatalytic CH_4_ oxidation by O_2_

Atomic Pd cocatalyst was prepared by the in situ photo-deposition method using K_2_PdCl_4_ and (NH_4_)_2_PdCl_4_ as the precursors on the visible-responsive In_2_O_3_ nanorod photocatalyst. Two types of photocatalysts were synthesized, including the defect-rich and defect-lean materials, denoted Pd-def-In_2_O_3_ and Pd-In_2_O_3_, respectively. For a comparison, other noble metals including Pt and Au modified photocatalysts were also prepared with the same dosage, denoted M-(def)-In_2_O_3_ (M = Pt and Au). With different K_2_PdCl_4_ dosage, the as-prepared samples were named as Pd_x_-def-In_2_O_3_, where x% represented the dosage weight percentage of Pd to In_2_O_3_. The real content of Pd in all photocatalysts was analyzed and shown in Table S1, which is very close to the nominal dosage. In the following discussion, the best sample Pd-def-In_2_O_3_ and the reference Pd-In_2_O_3_ were referred to Pd_0.1_-def-In_2_O_3_ and Pd_0.1_-In_2_O_3_ unless otherwise specified.

**Fig. 1:**
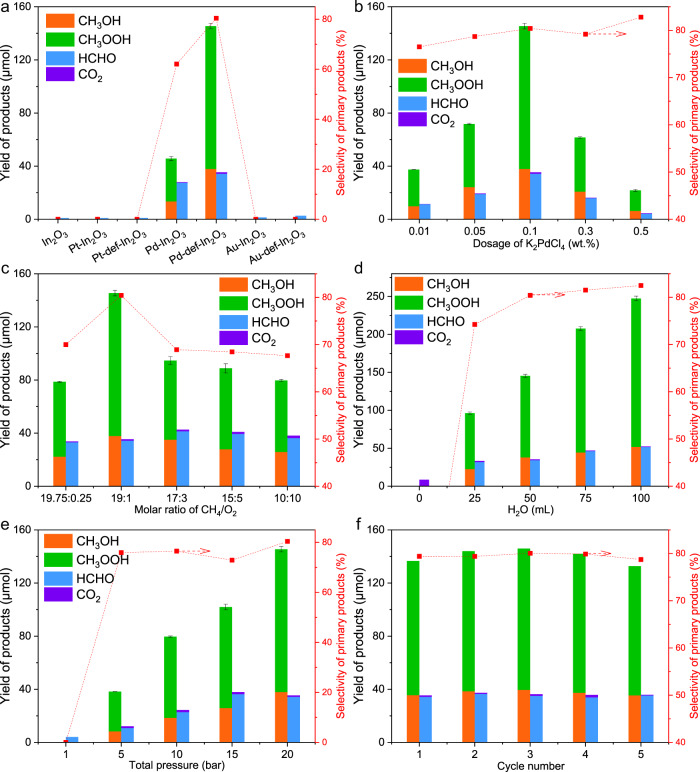
Photocatalytic CH_4_ conversion performance under 420 nm irradiation. Investigations on **a** diverse noble metal species, **b** K_2_PdCl_4_ dosage during synthesis, **c** molar ratio of CH_4_/O_2_, **d** H_2_O dosage, **e** total pressure and **f** cycling tests over the best sample Pd-def-In_2_O_3_. Standard reaction conditions: 20 mg photocatalyst, 50 ml distilled H_2_O, 19 bar CH_4_, 1 bar O_2_, 3 h. For reaction condition investigation, only the specified parameter was changed.

Typical noble metal cocatalysts (Pt, Pd, Au) loaded on In_2_O_3_ nanorods were first tested via photocatalytic CH_4_ conversion with O_2_ as the oxidant (Fig. 1a and Table S2). Under 420 nm irradiation, the products including CH_3_OH, CH_3_OOH and HCHO over Pd-In_2_O_3_ reaches 13.4, 32.3 and 27.5 μmol in 3 h reaction, respectively. The selectivity of the primary products (CH_3_OH and CH_3_OOH) was 62.1% and the selectivity to the overoxidation products (HCHO and CO_2_) was 37.9%. In a comparison, Au-In_2_O_3_ and Pt-In_2_O_3_ performed almost 100% over-oxidized products (HCHO), while with the trace yields of 1.4 and 0.9 μmol HCHO, respectively. Such difference was probably caused by the intrinsic characteristics of different noble metals, such as higher dehydrogenation capability of Pd than Au^49,50^. Meanwhile, Pt and Au-loaded catalysts seemed to over-oxidize of the major product CH_3_OH to HCHO as 100% selectivity of HCHO over the Pt and Au-loaded catalysts was observed. One possible reason is due to the weak ability of Pt or Au to trap photoholes as Pt and Au were mainly reported as electrons acceptors^51–53^, as such photoholes left on the VB of In_2_O_3_ with a strong oxidation ability could over-oxidize the products to HCHO. Further introduction of defective sites to form Pd-def-In_2_O_3_ led to an improved oxygenates yield to 179.7 μmol, 2.5 times higher than that of Pd-In_2_O_3_ (73.2 μmol). In parallel, the selectivity of the primary products was improved from 62.1 to 80.4%, suggesting that deep-oxidation to HCHO and CO_2_ was greatly suppressed under the synergy of Pd single atoms and OVs. In the case of Au-def-In_2_O_3_ and Pt-def-In_2_O_3_, defect modification exhibited the similar phenomenon on promoting CH_4_ conversion although the yield was much lower than that achieved on the Pd modified photocatalyst. Apparent quantum efficiency (AQE) of the photocatalytic reaction was determined to be 2.89% at 420 nm for Pd-def-In_2_O_3_, further indicating the efficient visible-light-driven activity.

The effect of Pd single atoms was explored over Pd_x_-def-In_2_O_3_. As shown in Fig. 1b, Pd_x_-def-In_2_O_3_ photocatalysts exhibited much higher oxygenates production than that of the pristine In_2_O_3_. With the raising of K_2_PdCl_4_ dosage, the production of the liquid oxygenates exhibited the volcanic trend, increasing from 48.7 μmol on Pd_0.01_-def-In_2_O_3_ to 179.7 μmol on Pd_0.1_-def-In_2_O_3_. Furthermore, the increasing K_2_PdCl_4_ dosage resulted in the decreased photocatalytic performance. Notably, the selectivity exhibited slight improvement from 76.5 to 82.8%. With a close look at the above results, OVs and Pd cocatalyst played the synergistic role in optimizing the activity and selectivity for photocatalytic CH_4_ conversion.

Molar ratio of CH_4_ to O_2_ was tuned over Pd-def-In_2_O_3_ (Fig. 1c). The production of oxygenates demonstrated the volcanic trend again, with the highest oxygenate production and selectivity achieved at 1 bar O_2_ pressure. Reducing molar ratio of CH_4_ to O_2_ caused the decreased production to 116.0 μmol of primary products when CH_4_/O_2_ = 10/10, mainly ascribed to the decrease of CH_4_ concentration. In parallel, the increased concentration of O_2_ induced overoxidation and decreased selectivity of the primary products from 80.4 to 67.6 %. With the increase of H_2_O dosage (Fig. 1d), the production of oxygenates gradually increased, reaching the highest value with 100 ml H_2_O dosage. The highest oxygenates achieved 299.0 μmol, 2.3 times improvement than that of 25 ml dosage (128.0 μmol) over Pd-def-In_2_O_3_. Moreover, the selectivity of the primary products improved from 74.2 to 82.5% with H_2_O dosage increasing from 25 to 100 ml, which could be attributed to the enhanced desorption of the products from the surface of the photocatalyst when more water was used as discussed later. Notably, in the absence of H_2_O dosage, CO_2_ (8.5 μmol) was produced as the only product, suggesting the critical role of H_2_O in promoting CH_4_ activation as well suppressing overoxidation, probably ascribed to the production of ·OH radical and promotion desorption of oxygenates by H_2_O^54^. While increasing the total pressure of the gaseous reactants, CH_4_ dissolved increased and the oxygenate production gradually increased (Fig. 1e), e.g., only trace amount of HCHO (4.1 μmol) produced at 1 bar and reaching the highest yield of 179.7 μmol when the pressure was 20 bar. To investigate the stability of the optimized photocatalyst, we carried out the cycling test experiment over Pd-def-In_2_O_3_ photocatalyst. No obvious decrease of oxygenates was observed under 15 h reaction (Fig. 1f), demonstrating the good stability of Pd-def-In_2_O_3_.

### Structural identification

X-ray diffraction (XRD) patterns were recorded to probe the crystalline structure of the representative photocatalysts (In_2_O_3_, Pd-In_2_O_3_ and Pd-def-In_2_O_3_) (Fig. S1). The diffraction peaks on all three samples at 30.7°, 35.5°, 51.0° and 60.7° were well matched with the standard phase of In_2_O_3_ (PDF#71-2194). While no Pd and PdO_x_ diffraction peaks were observed on Pd-In_2_O_3_ and Pd-def-In_2_O_3_, indicating the high dispersion of Pd species. The slightly weakened relative intensity from 100% of In_2_O_3_ to 97% and 93% of Pd-In_2_O_3_ and Pd-def-In_2_O_3_ could be probably ascribed to the introduction of defects. Raman spectra (Fig. S2) further supported the well-established In_2_O_3_ phase. The typical Raman peaks for In_2_O_3_ were clearly observed at 130.6, 305.1 and 494.8 cm^−1 ^^55^. For Pd-In_2_O_3_ and Pd-def-In_2_O_3_, the dominant peak exhibited a slight left-shift from 130.6 to 129.9 cm^−1^, attributed to the surface stain effect induced by the Pd cocatalyst deposition^56^.

**Fig. 2:**
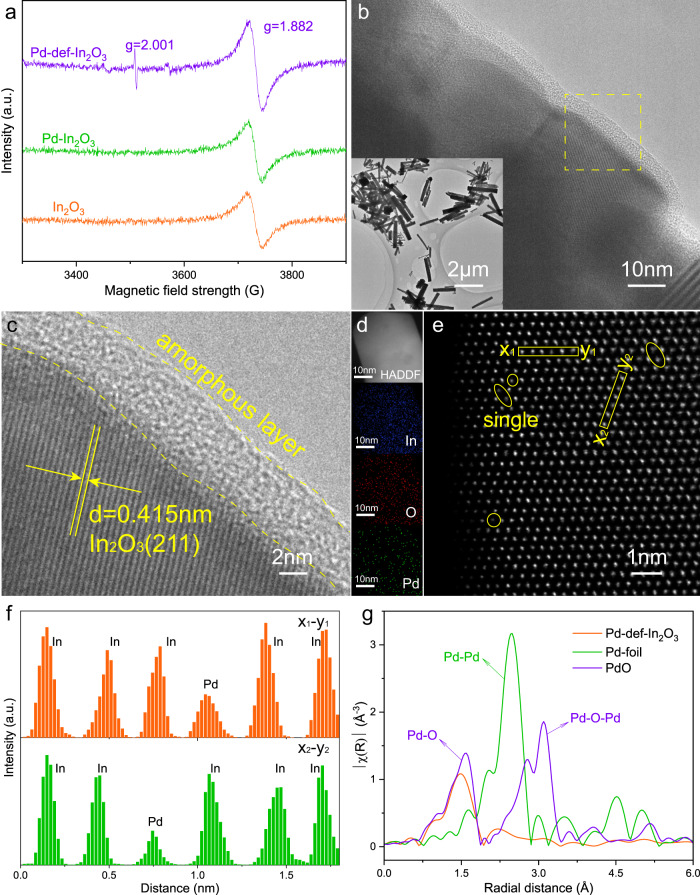
Structural identification. **a** EPR spectra of In_2_O_3_, Pd-In_2_O_3_ and Pd-def-In_2_O_3_. **b**, **c** HRTEM and **d** HAADF and EDS-mapping images of Pd-def-In_2_O_3_. Blue, red and green colors represent indium, oxygen and palladium elements, respectively. **e** Aberration corrected HAADF-STEM image of Pd-def-In_2_O_3,_ where Pd single atoms with a weak intensity are indicated by yellow circles. **f** Line scan measured along the x-y rectangle region marked in **e**. **g** Fourier transforms of EXAFS of the Pd K-edge spectra of Pd-def-In_2_O_3_, Pd foil and PdO.

Electron paramagnetic resonance (EPR) spectra were conducted to evaluate the spin electrons including OVs (Fig. 2a). For the pristine In_2_O_3_ and Pd-In_2_O_3_, a single Lorentz peak at *g* = 1.882 was observed, ascribed to the electrons on the conduction band (CB)^57,58^. In the case of Pd-def-In_2_O_3_, the signal of this peak exhibited much stronger intensity than the others, suggesting the higher electron density on CB. Meanwhile, an additional Lorentz peak was observed at *g* = 2.001, which could be attributed to the free-electrons trapped by the OVs^58^, thus suggesting the existence of OVs in Pd-def-In_2_O_3_. High-resolution O1s XPS measurement of Pd-def-In_2_O_3_ photocatalyst was then conducted and shown in Fig. S3a. The binding peaks centered at 529.72 eV, 531.59 eV and 533.02 eV were assigned to the lattice oxygen, OVs and adsorbed moisture, respectively, which directly supported the existence of OVs, consistent with the EPR results and amorphous layer discussed below^59^. In comparison, Pd-In_2_O_3_ (Fig. S3b) exhibited a relatively low content of 14% at 531.59 eV, much lower than that of Pd-def-In_2_O_3_ (30%).

High-resolution transmission electron microscope (HRTEM) images further proved the defective structure of Pd-def-In_2_O_3_ (Fig. 2b, c). Pd-def-In_2_O_3_ reserved the nanorod morphology with the average dimension of 240 nm in diameter and 1449 nm in length (insert of Figs. 2b and S4), the same as that of the In_2_O_3_ substrate and Pd-In_2_O_3_. In addition, a thin amorphous/defective layer of ca. 4 nm was observed on the edge (Fig. 2c). The bulk crystal plane distance of 0.415 nm was indexed to the (211) facet of In_2_O_3_. On the contrary, there is not such defective layer as indicated in Fig. S5. Elemental distribution of the corresponding area was analyzed by the EDS-mapping images (Fig. 2d). Obviously, Pd-def-In_2_O_3_ exhibited uniform palladium distribution with indium and oxygen elements. No Pd nanoparticles or clusters were found from the HAADF-STEM images at different scales apart from the 42 nm In_2_O_3_-assembled nanorods (Fig. S4). The aberration corrected HAADF-STEM image in Fig. 2e clearly indicated the atomic distribution of Pd, where the weak intensity spots cycled by the yellow corresponded to Pd atoms. The x-y line scan along the yellow rectangle of Fig. 2e clearly presents the atomic dispersion of Pd as shown in Fig. 2f. To further investigate the coordination environment of Pd species, Pd K-edge X-ray absorption near edge structure spectra of Pd-def-In_2_O_3_ with Pd foil and PdO references were measured as shown in Fig. S6. The absorption edge position of Pd-def-In_2_O_3_ was observed between Pd foil and PdO, suggesting the valence state of Pd in Pd-def-In_2_O_3_ was between 0 and +2^60^. Fourier transforms of EXAFS of the Pd K-edge (Fig. 2g) exhibited only a predominant peak at ca. 1.5 Å for Pd-def-In_2_O_3_, which was ascribed to the first-shell of the Pd-O bond with reference to the PdO sample. In parallel, no peaks corresponding to Pd-O-Pd and Pd-Pd at 3.1 Å and 2.5 Å were detected, indicating the atomically dispersed Pd sites in Pd-def-In_2_O_3_, with the absence of metallic Pd and PdO clusters. Therefore the best sample was composed of single atom Pd and OVs on In_2_O_3_ nanorods.

### Mechanism investigation

**Fig. 3:**
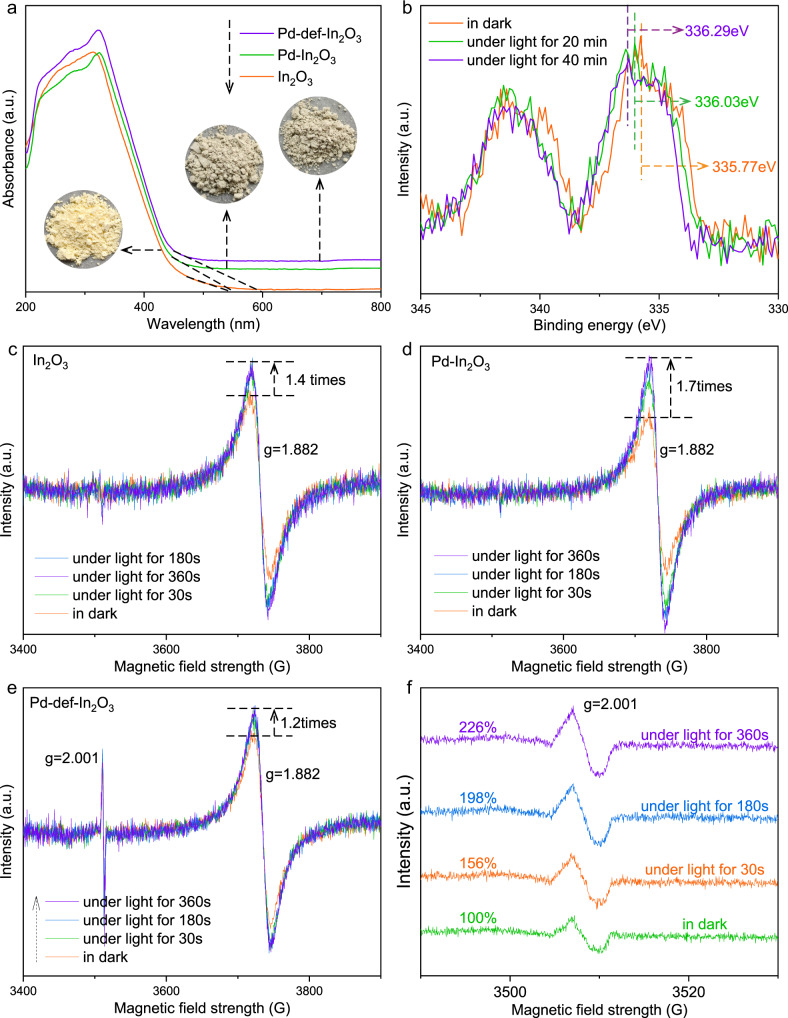
Photo-physical properties. **a** UV-DRS spectra of In_2_O_3_, Pd-In_2_O_3_ and Pd-def-In_2_O_3_. Insert shows the optical images of In_2_O_3_, Pd-In_2_O_3_ and Pd-def-In_2_O_3_. **b** In situ Pd_3d_ XPS spectra of Pd_0.3_-def-In_2_O_3_ in dark and under extended light irradiation. Solid-state in situ EPR spectra of **c** In_2_O_3_, **d** Pd-In_2_O_3_ and **e**, **f** Pd-def-In_2_O_3_ in dark and under light irradiation for different reaction time.

UV-vis diffuse reflection spectra (UV-DRS) were conducted to evaluate the photoabsorption ability of the representative photocatalysts (In_2_O_3_, Pd-In_2_O_3_ and Pd-def-In_2_O_3_) (Fig. 3a). All three photocatalysts exhibited the similar band photoabsorption onset at ca. 450 nm, indicating that the modification of single atom Pd and OVs had little influence on the bandgap energy, which would not be the decisive reason for the induced improvement of photocatalysis. Meanwhile, the intrinsic defective structure in the nanocrystallines led to the tail adsorption to 560 nm, and the introduction of OVs resulted in a further red-shift of this tail to 600 nm, similar to the reported on TiO_2_^61^. Meanwhile, since Pd exhibited the atomic dispersion but not metal nanoparticles, it thus could not cause a photoabsorption peak via the localized surface plasmon resonance effect^62^.

In situ high-resolution Pd_3d_ X-ray photoelectric spectra (XPS) in dark and under light irradiation were then conducted to investigate the charge transfer direction (Figs. 3b and S7). Notably, high-resolution Pd_3d_ XPS spectra of Pd_0.1_-def-In_2_O_3_ showed a very weak Pd signal (Fig. S8), centered at ca. 336.0 eV between the binding energy of Pd^0^ (335.38 eV) and Pd^2+^ (336.55 eV)^63^. The de-convoluted peaks of Pd^0^ and Pd^2+^ at 335.38 and 336.55 eV showed the relative content of 58% and 42%, respectively, indicating that the Pd valance states were equivalent to a mixture of Pd^0^ and Pd^2+^. However, due to the limit detection sensitivity of the in situ XPS spectra (Thermo ESCALAB 250Xi instrument), the in situ XPS under light irradiation for Pd_0.1_-def-In_2_O_3_ was featureless as shown in Fig. S9. Therefore, Pd_0.3_-def-In_2_O_3_ that prepared under the identical conditions but with a higher content of Pd (0.284 wt%) was employed to study the function of Pd through the in situ XPS measurement (Fig. 3b). The oxidation state for Pd_0.3_-def-In_2_O_3_ in dark (Fig. 3b) was mainly close to Pd^0^, suggesting that Pd aggregation happened with a high dosage of K_2_PdCl_4_ precursor. It could be seen that Pd_3d_ XPS spectra exhibited an attractive phenomenon that a left-shift of the strongest peaks was observed from 335.77 eV in dark to 336.03 eV for 20 min irradiation and to 336.29 eV for 40 min irradiation. Further extending irradiation time could not result into a continuous left-shift when comparing the XPS peaks under light irradiation for 40 and 60 min (Fig. S7). Such left shift to higher binding energy indicated a more positive valence of Pd under light irradiation, thus suggesting the role of Pd as the hole acceptor under light irradiation. The solid-state in situ EPR spectra under light irradiation provided further experimental evidence as shown in Fig. 3c–f. For pristine In_2_O_3_, EPR signal at *g* = 1.882 (Fig. 3c) was attributed to the spin electrons on the CB of In_2_O_3_ substrates^57,58^. Under light irradiation, this signal for In_2_O_3_ showed an increased intensity by 1.4 times due to the photo-excitation process. Similarly, Pd-In_2_O_3_ also exhibited such EPR signal that associated with the CB electrons at *g* = 1.882, and an enhanced intensity under light irradiation. However, the enhancement of this EPR signal for Pd-In_2_O_3_ was about 1.7 times, much larger than that of In_2_O_3_ (1.4 times) (Fig. 3d). Such greatly enhanced intensity thus suggested that Pd could accept photo-induced holes, then leading to the suppressed charge recombination and an improved concentration of spin electrons that were photo-excited to the CB.

In situ EPR spectra under light irradiation also gave information on the role of OVs. Under light irradiation for 30 s, the EPR signal intensity for Pd-def-In_2_O_3_ at *g* = 1.882 (Fig. 3e) that associated with the spin electrons on the CB increased by 1.2 times. Further prolonging irradiation time did not result in continuous increased EPR intensity at *g* = 1.882, suggesting that dynamic equilibrium between photo-excitation and charge transfer quickly reached. In parallel, the related signal at *g* = 2.001 that assigned to the OVs performed gradually increasing intensity from 100 to 226% (Fig. 3f) with the prolonged irradiation for 360 s. Such enhanced intensity indicated the role of OVs as electron acceptors and continuous electron transfer from the CB to the OVs. Therefore, single atom Pd and OVs separately acted as the hole and electron acceptors under light irradiation, which would greatly contribute to the enhanced charge separation.

Photocurrent responses (Fig. S10) were tested to evaluate the charge separation efficiency. Pristine In_2_O_3_ exhibited a relatively low photocurrent density of 61.8 μA cm^−2^. After photo-depositing high dispersed Pd cocatalyst, Pd-In_2_O_3_ nearly doubled photocurrent density to 120.6 μA cm^−2^. The photocurrent density was further improved to 168.1 μA cm^−2^ on Pd-def-In_2_O_3_, almost 2.7 and 1.4 times enhancement than that of In_2_O_3_ and Pd-In_2_O_3_, respectively. Such highest photocurrent density on Pd-def-In_2_O_3_ attributed to the most efficient charge separation, indicating the defects and single atom Pd could greatly enhance charge transfer, which is consistent with the analysis mentioned above. Steady-state fluorescence (PL) spectra further evidenced the enhanced charge separation efficiency. As shown in Fig. S11, a relatively strong PL emission peak was observed for the pristine In_2_O_3_, attributed to the severe charge recombination. In comparison, the PL intensity for Pd-In_2_O_3_ was greatly weakened, indicating the suppressed charge recombination. For Pd-def-In_2_O_3_ photocatalyst, the most weakened PL peak were observed, ascribed to the most enhanced charge separation efficiency, which was corresponding with the photocurrent analysis. Time-decay PL spectra were conducted to evaluate the PL lifetime. As shown in Fig. S12, Pd-def-In_2_O_3_ photocatalyst exhibited the slowest PL decay kinetics. The fitting results (Table S3) showed that Pd-def-In_2_O_3_ exhibited the average PL lifetime at 4.99 ns, longer than that of In_2_O_3_ (3.60 ns) and Pd-In_2_O_3_ (4.28 ns), which would be beneficial to the efficient utilization of separated charge carriers.

**Fig. 4:**
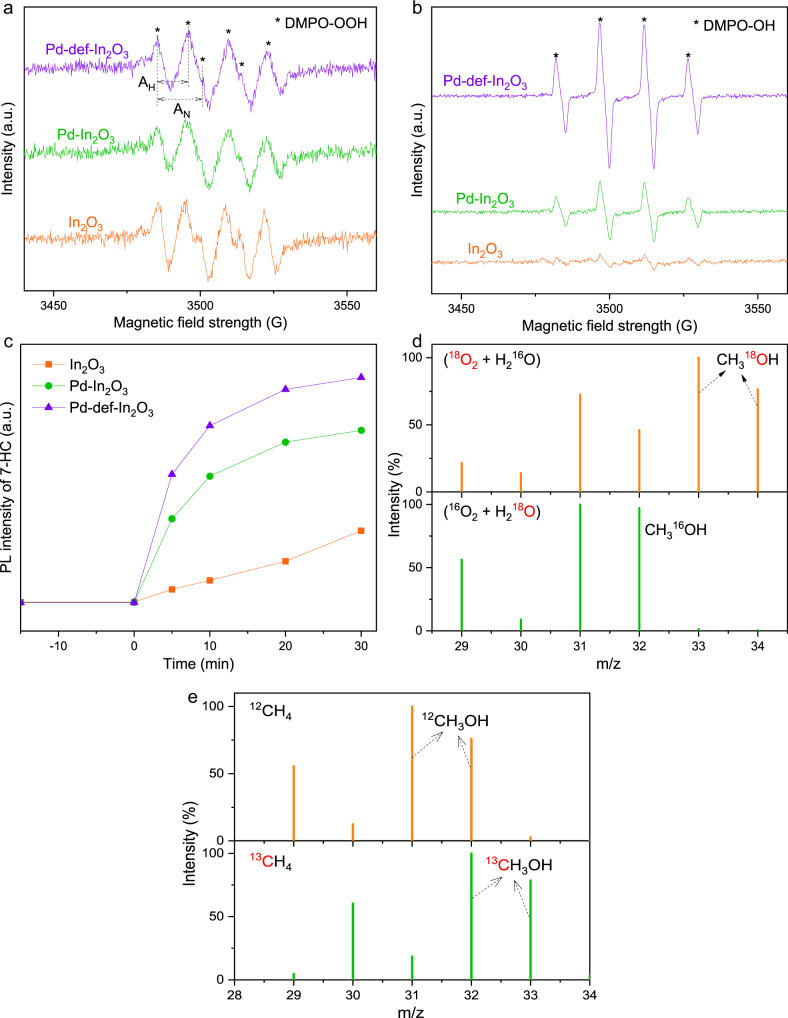
Photo-chemical processes. In situ EPR spectra of **a** DMPO-OOH and **b** DMPO-OH for the observation of reactive ·OOH and ·OH radicals over different photocatalysts. A_H_ and A_N_ were hyperfine coupling constants of hydrogen and nitrogen elements, respectively. **c** PL intensity of 7-HC vs. time over different photocatalysts for the quantification of ·OH. Isotopic labeled experiments **d** oxygen source and **e** carbon source for methanol production in the presence of isotopic labeled H_2_^18^O, ^18^O_2_ or ^13^CH_4_.

ROS including ·OOH and ·OH radicals were widely regarded as the main active species for CH_4_ activation^64^ and monitored by in situ EPR spectra with 5, 5-dimethyl-1-pyrroline N-oxide (DMPO) as the spin-electron trapping agents. As shown in Fig. 4a, six prominent characteristic signals (4 strong and 2 relatively weak peaks) with the hyperfine splitting constants measured as A_N_ = 15.4 G and A_H_ = 10.6 G were clearly observed and attributed to the DMPO-OOH adduct^65^. Such detected ·OOH radicals over different photocatalysts came from the reduction of O_2_ molecule with photo-induced electrons and H^+^. A stronger intensity of DMPO-OOH was observed for Pd-def-In_2_O_3_, suggesting the production of ·OOH radical was enhanced by the integration of single atom Pd and OVs. On the other hand, in situ EPR spectra under light irradiation was used to monitor the generation of ·OH radical with DMPO as the trapping agent in H_2_O. The 1:2:2:1 quartet signal was observed and assigned to the DMPO-OH adduct, suggesting the generation of ·OH radical (Fig. 4b). It was obvious that Pd-def-In_2_O_3_ produced much more ·OH under identical conditions than Pd-In_2_O_3_ and In_2_O_3_ was the worse. It is believed that ·OH initially activates CH_4_ to methyl radical (·CH_3_), thus Pd-def-In_2_O_3_ performed CH_4_ activation best followed by Pd-In_2_O_3_, which is consistent with the step by step enhanced photocatalytic performances by Pd and then both Pd and OVs, indicating that OVs could promote charge separation and also facilitate water oxidation reaction on Pd. Coumarin was used as the probe for ·OH radical detection due to the easy reaction between coumarin and ·OH to produce 7-hydroxycoumain (7-HC) that could be detected by UV-vis spectra at 412 nm (Fig. 4c). The results further supported that Pd-def-In_2_O_3_ held the strongest ability for ·OH production, which facilitated CH_4_ activation. Therefore, single atom Pd worked as the hole acceptor, which then catalyzed ·OH radical production from water oxidation. Simultaneously, OVs acted as the electron acceptor, which then catalyzed O_2_ reduction to generate ·OOH radical.

The reaction pathway was investigated by isotopic labeled experiments, including using H_2_^18^O and ^18^O_2_. In the presence of 3 ml H_2_^18^O, 1 bar ^16^O_2_ and 19 bar CH_4_, no isotopic labeled CH_3_^18^OH (*m*/*z* = 33 and 34) was detected by GCMS (Fig. 4d), suggesting H_2_O was not the oxygen source that directly participated the formation of oxygenates. In parallel, when using 3 ml H_2_^16^O, 1 bar ^18^O_2_ and 19 bar CH_4_, the signals at *m*/*z* = 34 and 33 were attributed to the isotopic labeled CH_3_^18^OH and its fragment (Fig. 4d), suggesting O_2_ was the only oxygen source for CH_3_OH formation. Carbon source for methanol production were also studied in the presence of 5 bar isotopic labeled ^13^CH_4_ (Fig. 4e), where the signal of mass spectra (MS) at *m*/*z* = 33 was ascribed to ^13^CH_3_OH, demonstrating that CH_4_ was the carbon source for oxygenates production.

**Fig. 5:**
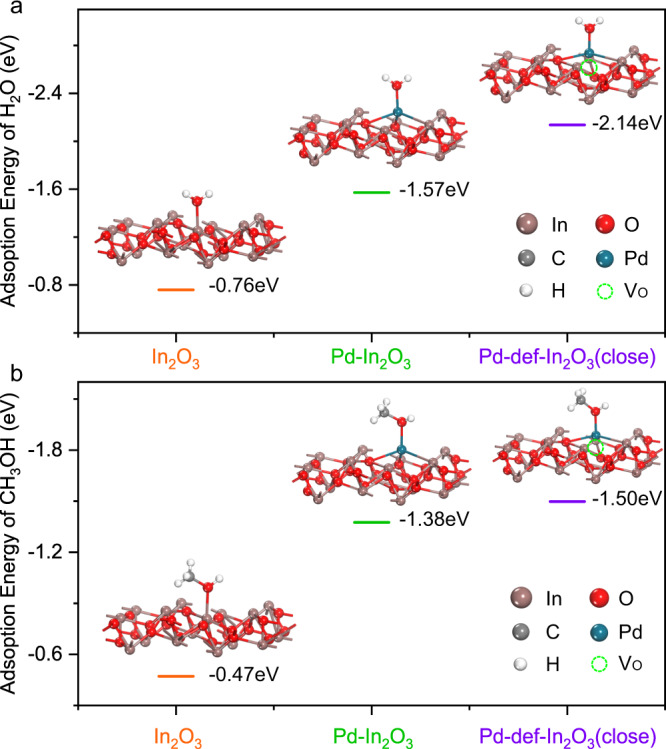
DFT calculation of optimized geometries and adsorption energies. **a** H_2_O and **b** CH_3_OH on In_2_O_3_, Pd-In_2_O_3_ and Pd-def-In_2_O_3_(close).

DFT calculations (Fig. 5) were conducted to explain the improved selectivity of primary products. It should be noted that timely desorption of the primary products on the active sites could efficiently avoid its deep-oxidation to HCHO and CO_2_. As ·OH radical was regarded as the main species that induced oxidation on single atom Pd cocatalyst, it was accordingly considered that the efficient desorption of primary products like CH_3_OH on Pd is critical to suppress further oxidation. Four models including In_2_O_3_ with (111) facet, Pd-In_2_O_3_, Pd-def-In_2_O_3_(close) with one OV close to single atom Pd and Pd-def-In_2_O_3_(far) with one OV far away from the single atom Pd modification were built. Then the adsorption energies of H_2_O and CH_3_OH on these models were calculated by the density functional theory (DFT) since the strong adsorption of H_2_O might promote the desorption of CH_3_OH. As shown in Figs. 5a, b and S13, the adsorption energies of H_2_O on In_2_O_3_, Pd-In_2_O_3_, Pd-def-In_2_O_3_(far) and Pd-def-In_2_O_3_(close) were −0.76, −1.57, −1.76 and −2.14 eV, respectively, much larger than the CH_3_OH adsorption energy of −0.47, −1.38, −1.40 and −1.50 eV on the specified model. Such large adsorption energies indicate CH_3_OH could be easily replaced by H_2_O on In_2_O_3_ or Pd atoms, promoting the desorption of CH_3_OH. Moreover, adsorption energies of water further increased with the introduction of both Pd atom and OV, demonstrating such co-modification of Pd atoms and OVs could promote the adsorption of H_2_O most efficiently, which was consistent with the increased production of ·OH radicals as analyzed by the in situ EPR and coumarin experiments. Though the adsorption of CH_3_OH was also enhanced due to the introduction of Pd atoms and OVs, H_2_O adsorption energy was enhanced much more, thus water could facilitate the desorption of primary products and avoid overoxidation as indicated in Fig. 1d. It also suggested that a close distance of Pd with OVs was preferable for the competitive adsorption of H_2_O, thus facilitating the desorption of CH_3_OH.

**Fig. 6:**
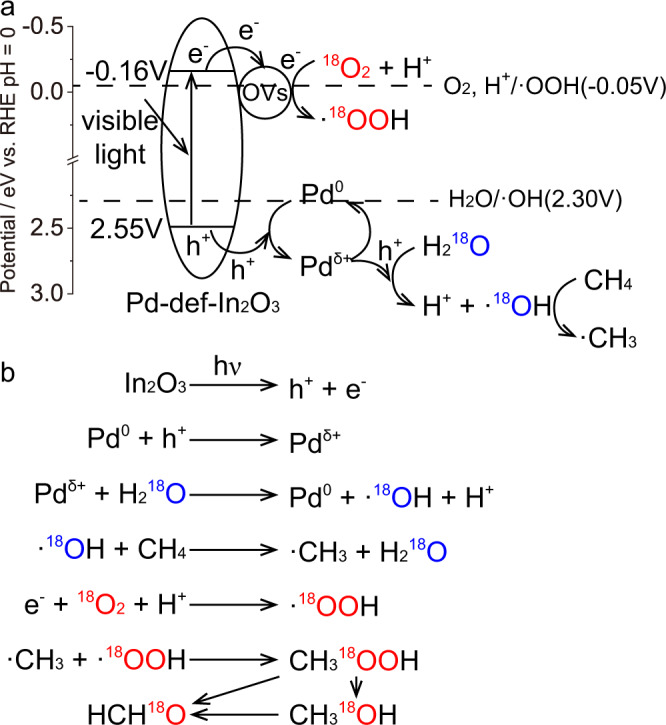
Illustration of aerobic photocatalytic CH_4_ conversion over Pd-def-In_2_O_3_. **a** Charge transfer and separation induced by Pd and oxygen vacancies. **b** The oxygenate production with O_2_ as the only oxygen source and H_2_O as the promoter for CH_4_ activation.

Based on the above results, aerobic photocatalytic CH_4_ conversion mechanism over Pd-def-In_2_O_3_ was proposed (Fig. 6). The bandgap structure of Pd-def-In_2_O_3_ was established in terms of UV-DRS spectra (Fig. 3a) and Mott-Schottky plots (Fig. S14), where the calculated bandgap energy, the CB position and the valence band position were determined to be 2.71 eV, −0.16 V and 2.55 V (vs. RHE), respectively, which is sufficient to drive O_2_ reduction to produce ·OOH radicals and H_2_O oxidation to produce ·OH radicals. Compared with the widely reported large bandgap semiconductor photocatalysts, e.g., TiO_2_, this relatively weak oxidation potential mitigated the potential to overoxidation. The exact energy level of OVs in the optimized catalyst was hard to be established probably due to its low content. Generally, under 420 nm irradiation, electrons (e^−^) were excited to the CB of In_2_O_3_ nanorod photocatalyst, while leaving holes (h^+^) on the valence band. Then the photo-induced electrons were trapped by the OVs, activating O_2_ with H^+^ to produced ·OOH radicals as detected by the in situ EPR spectra. In parallel, Pd atoms served as the hole acceptors (Pd + h^+^ → Pd^δ+^), and then reacted with the adsorbed H_2_O to produced ·OH (Pd^δ+^ + H_2_O → Pd^0^ + ·OH + H^+^). CH_4_ molecules were next activated by the as-produced ·OH to ·CH_3_. The coupling reaction between ·CH_3_ and ·OOH then generated the primary products (CH_3_OOH), and subsequently transferred to CH_3_OH as indicated in Fig. 6b. Compared with the pristine In_2_O_3_, the incorporation of Pd single atoms significantly promoted charge separation and facilitated the generation of reactive species, thus promoting CH_4_ conversion to oxygenates. Pd atoms loading also moderated the oxidation ability of photoinduced holes on In_2_O_3_ as indicated in Fig. 6a, reducing the overoxidation of the primary products. Further decoration of OVs could strengthen the promoted charge separation efficiency, which eventually resulted in the superior photocatalytic CH_4_ conversion activity and selectivity. In order to suppress overoxidation, it was also critical to enhance the desorption of primary oxygenate products by H_2_O, as supported by the DFT calculation.

In summary, visible-light-driven CH_4_ conversion at ambient temperature was reported over the In_2_O_3_ nanorod photocatalyst loaded by Pd single atoms cocatalysts and OVs. Under 420 nm irradiation, superior yield (99.7 μmol h^−1^) and selectivity (82.5%) of the primary products were achieved on the Pd-def-In_2_O_3_ photocatalyst under optimized reaction conditions. In situ XPS and EPR spectra under visible light irradiation indicated that Pd and OVs acted as the hole and electron acceptors, respectively, thus synergistically boosting charge separation and transfer. Isotopic labeled experiments proved that O_2_ was the only oxygen source for oxygenates production, while H_2_O was the promoter of CH_4_ activation through the production of ·OH radical as monitored by the in situ EPR spectra with DMPO as the spin-trapping agent. DFT calculation results suggested that H_2_O performed much larger adsorption energies than CH_3_OH on either In_2_O_3_, def-In_2_O_3_ or Pd-def-In_2_O_3_, suggesting the stronger adsorption of H_2_O than CH_3_OH, which was beneficial to the timely desorption of CH_3_OH produced, thus avoiding further overoxidation. The introduction of Pd and OVs could further improve the selectivity of primary oxygenates mainly through the greatly enhanced adsorption of H_2_O and the reduced oxidation potential of photoinduced holes. This work thus provided a useful avenue on co-modification of photocatalysts by oxidative single atom cocatalyst and OVs for simultaneous regulation of both activity and selectivity through enhancing charge separation, moderating photohole oxidation ability and timely promoting desorption of primary products by a solvent.

## Methods

### Synthesis of In_2_O_3_ nanorods

In_2_O_3_ nanorods were prepared according to the previous study^66^. Typically, 12.0 g urea and 1.5 g InCl_3_ were dissolved in 135 g H_2_O, followed by stirring at 80 °C for 14 h. After naturally cooling down, the reactant was centrifuged and washed with H_2_O for several times. The white powder was then dried in vacuum at 60 °C overnight. After thermal-treating in air at 700 °C for 5 h at a ramping rate of 5 °C min^−1^, yellow powder was obtained and denoted as In_2_O_3_.

### Synthesis of Pd_x_-In_2_O_3_ and Pd_x_-def-In_2_O_3_ nanorods

Pd_x_-In_2_O_3_ and Pd_x_-def-In_2_O_3_ nanorods were prepared through photo-deposition with ammonium tetrachloropalladate(II) ((NH_4_)_2_PdCl_4_) and potassium tetrachloropalladate(II) (K_2_PdCl_4_) as the precursors, respectively. The synthesis was conducted in the multichannel reactor (Beijing Perfectlight Technology Co., Ltd). For Pd_x_-def-In_2_O_3_ preparation, 200 mg In_2_O_3_ was first dispersed through sonication with the aqueous solution containing 10 vol.% methanol. Then certain amount of K_2_PdCl_4_ solution was added. After sealing and purging with ultrapure argon (99.999 vol.%) for 30 min, the reactor was bottom-irradiated for 3 h to facilitate Pd photo-deposition. The suspension was then centrifuged, washed with H_2_O for several times and dried under vacuum at 60 °C overnight. The as-prepared samples were denoted as Pd_x_-def-In_2_O_3_, where x% represented the mass percentage of palladium to In_2_O_3_ substrates. OVs in Pd_x_-def-In_2_O_3_ were probably generated due to the quite acidic condition of K_2_PdCl_4_ solution with a pH value of 3.84, lower than that of (NH_4_)_2_PdCl_4_ solution with pH = 4.20. Pd_x_-In_2_O_3_ with no OVs was prepared under identical conditions except the usage of (NH_4_)_2_PdCl_4_ instead of K_2_PdCl_4_.

### Characterizations

XRD were measured to obtain the crystalline structure on the D8 ADVANCE diffractometer (Bruker Co., Ltd). Palladium and potassium contents were measured through inductively coupled plasma optical emission spectrometer (ICP-OES) (Agilent 5110 ICP-OES/MS). Chlorine contents in the photocatalyst were analyzed by the ion chromatography (ICS-5000). Both K^+^ and Cl^-^ ions did not exist in the as-prepared photocatalyst according to the measurements (Table S1). Raman spectra were collected on the DXR 2DXR2 instrument (Thermo Fisher Scientific, Co., Ltd). HRTEM images were captured on the Talos F200X instrument (FEI Co., Ltd). UV-DRS spectra were measured on the UV-3600 plus spectrophotometer (Shimadzu Co., Ltd). The extend X-ray absorption fine structure (EXAFS) spectra were collected at BL14W1 station in Shanghai Synchrotron Radiation Facility. Photocurrent test was conducted on the electronic workstation (CHI660E) on the three-electrode system. Ag/AgCl electrode, platinum sheet electrode and Na_2_SO_4_ solution (0.1 M) were used as the reference electrode, counter electrode and electrolyte, respectively. The mixtures of photocatalyst, ethanol and Nifion solution (Shanghai Adamas Reagent Co., Ltd) were suspended and sonicated to prepare the working electrode. In situ XPS in dark and under light irradiation for different irradiation time were measured on the Thermo ESCALAB 250Xi instrument with an Al Kα radiation source. In situ solid-state EPR spectra in dark and under light irradiation were measured at room temperature with 20 mg photocatalyst on the ELEXSYS II EPR instrument.

### Photocatalytic CH_4_ conversion

Photocatalytic CH_4_ conversion was conducted in a top-irradiated high-pressure reactor with 200 ml volume. LED lamp (420 nm, PLS-LED100C, Beijing Perfectlight Technology Co., Ltd) was used as the light source. Radiation spectrum of the LED lamp contained only visible light centering at 421 ± 7 nm (Fig. S15). Typically, 20 mg photocatalyst was dispersed in 50 ml distilled water. After sealing and purging with ultrapure O_2_ (99.999 vol.%) for 20 min, 1 bar O_2_ and 19 bar CH_4_ (99.999 vol.%) were flowed into the reactor. The temperature of the reactor was maintained at 25 °C by the cold-water bath. After reacting for 3 h, the gaseous and liquid products were measured by the gas chromatography (GC2014, Shimadzu Co., Ltd) equipped with thermal conductivity detector and flame ionization detector, which showed that no CO product was detected for all of the photocatalytic reactions under the present experimental conditions. CH_3_OOH was measured through ^1^H nuclear magnetic resonance (NMR) (AVANCE III, JEOL Ltd). As CH_3_OOH and CH_3_OH have the same number of methyl, the area ratio of CH_3_OOH to CH_3_OH in ^1^H NMR should be the molar ratio of CH_3_OOH to CH_3_OH. Thus, CH_3_OOH was quantified with ^1^H NMR. The representative GC and NMR curves were shown in Figs. S16 and S17. HCHO was measured through the colorimetric method on the UV-3600 Plus spectrometer (Shimadzu Co., Ltd)^67^.

AQE was then measured. In the experiment, 100 mg Pd-def-In_2_O_3_ photocatalyst was first dispersed in 100 ml distilled water. Then the suspension was stirred and purged with ultrapure O_2_ (99.999 vol.%) for 20 min. After flowing 1 bar O_2_ and 19 bar CH_4_ into the reactor, the reaction was conducted for 2 h with a Xe lamp as the light source equipped with a 420 nm bandpass filter. Light intensity was measured as 36.6 mW cm^−2^ by the light intensity meter (PL-MW2000, Beijing Perfectlight Technology Co., Ltd). The as-produced CH_3_OOH, CH_3_OH and HCHO were 19.1, 7.6 and 8.4 μmol, respectively. Since the formations of CH_3_OOH, CH_3_OH and HCHO needed 1, 3 and 5 photogenerated charges^1^, respectively, AQE was calculated based on the following equation:

1 $${{{{{\rm{AQE}}}}}} =	\,\frac{{{{{{\rm{{Number}}}}}}}\,{{{{{{\rm{of}}}}}}}\,{{{{{{\rm{used}}}}}}}\,{{{{{{\rm{photons}}}}}}}}{{{{{{{\rm{Number}}}}}}}\,{{{{{{\rm{of}}}}}}}\,{{{{{{\rm{incident}}}}}}}\,{{{{{{\rm{photons}}}}}}}}\\ =	\,\frac{n\left({{{{{\rm{C}}}}}}{{{{{{\rm{H}}}}}}}_{3}{{{{{{\rm{OOH}}}}}}}\right)+n\left({{{{{\rm{C}}}}}}{{{{{{\rm{H}}}}}}}_{3}{{{{{{\rm{OH}}}}}}}\right)\times 3+n\left({{{{{{\rm{HCHO}}}}}}}\right)\times 5}{n({{{{{{\rm{photons}}}}}}})}$$

*n*(CH_3_OOH), *n*(CH_3_OH) and *n*(HCHO) represent the number of CH_3_OOH, CH_3_OH and HCHO, respectively, and *n*(photons) represents the number of the irradiated photons during methane conversion.

### Isotope labeling experiments

For carbon source investigation: 20 mg Pd-def-In_2_O_3_ photocatalyst was dispersed in 3 ml H_2_O. After the reactor being degassed for 30 min, 1 bar O_2_ and 5 bar ^13^CH_4_ were injected into the reactor. After reacting for 6 h, the suspension was filtered and then the solvent was analyzed by GC-MS (QP2020, Shimadzu Co., Ltd) equipped with the Cap WAX column.

For oxygen source investigation: 20 mg Pd-def-In_2_O_3_ photocatalyst was dispersed in 3 ml H_2_^16^O or H_2_^18^O. After the reactor being degassed for 30 min, 1 bar ^18^O_2_ or ^16^O_2_ and 5 bar CH_4_ were injected into the reactor. After reacting for 6 h, the suspension was filtered and then the solvent was analyzed by GC-MS (QP2020, Shimadzu Co., Ltd).

### Monitor of the reactive species

DMPO was used as the spin-trapping agent to monitor the reactive species including ·OOH and ·OH radicals. For ·OOH radical detection, 10 mg Pd-def-In_2_O_3_ photocatalyst was dispersed into 5 ml DMPO/methanol solution. After purging with ultrapure O_2_ (99.999 vol.%) for 20 min, in situ EPR spectra in dark and under light irradiation were collected. For ·OH radical detection, 10 mg Pd-def-In_2_O_3_ photocatalyst was dispersed in 5 ml aqueous DMPO solution. After purging with ultrapure O_2_ (99.999 vol.%) for 20 min, in situ EPR spectra in dark and under light irradiation were collected.

### Analysis of hydroxyl radical (·OH)

Coumarin was used as the probe for the quantification of ·OH via the production of 7-HC^41^. Typically, 20 mg photocatalyst was dispersed in 100 ml aqueous coumarin solution (5 × 10^−4^ M). After stirring for 30 min in dark, the suspension was irradiated with the LED light source (420 nm, PLS-LED100C, Beijing Perfectlight Technology Co., Ltd). Certain amount of suspension was sampled and filtered in the 10 min intervals. PL intensity of the produced 7-HC in the solution was then measured on the F4500 spectrofluorometer.

### DFT calculation of adsorption energies

The first-principles were employed to perform all the DFT calculations within the generalized gradient approximation using the PBE formulation. The projected augmented wave potentials have been chosen to describe the ionic cores and take valence electrons into account using a plane wave basis set with a kinetic energy cutoff of 400 eV. Partial occupancies of the Kohn–Sham orbitals were allowed using the Gaussian smearing method and a width of 0.05 eV. The electronic energy was considered self-consistent when the energy change was smaller than 10^−4^ eV. A geometry optimization was considered convergent when the force change was smaller than 0.05 eV/Å. Grimme’s DFT-D3 methodology was applied to describe the dispersion interactions. During structural optimizations, the 2 × 2 × 1 Monkhorst-Pack k-point grid for Brillouin zone was used for k-point sampling for structures. Finally, the adsorption energies (*E*_ads_) were calculated as *E*_ads_ = *E*_ad/sub_ − *E*_ad_ − *E*_sub_, where *E*_ad/sub_, *E*_ad_, and *E*_sub_ were the total energies of the optimized adsorbate/substrate system, the adsorbate in the structure, and the clean substrate, respectively.

## Supplementary information

Supplementary Information

## Data Availability

The data that support the findings of this study are available from the corresponding author upon reasonable request.
