# Supplementary material for: Synergy of Pd atoms and oxygen vacancies on In2O3 for methane conversion under visible light
Source: Nat Commun. 2022 May 25;13:2930. doi: 10.1038/s41467-022-30434-0 (PMC9132922; doi:10.1038/s41467-022-30434-0)
Supplement: Supplementary file 1 — Supplementary Information [file 41467_2022_30434_MOESM1_ESM.pdf]

Supporting Information

Synergy of Pd Atoms and Oxygen Vacancies on In_2O_3 for Methane Conversion under Visible Light

Lei Luo[1], Lei Fu[1], Huifen Liu[1], Youxun Xu[2], Jialiang Xing[1], Chun-Ran Chang[3], Dong-Yuan Yang[3, 4]*, Junwang Tang[2]*

[1] Key Lab of Synthetic and Natural Functional Molecule Chemistry of Ministry of Education, the Energy and Catalysis Hub, College of Chemistry and Materials Science, Northwest University, Xi'an, P. R. China. [2] Department of Chemical Engineering, University College London, Torrington Place, London WC1E 7JE, UK. [3] Shaanxi Key Laboratory of Energy Chemical Process Intensification, School of Chemical Engineering and Technology, Xi'an Jiaotong University, Xi'an, P. R. China. [4] Shaanxi yanchang Petroleum (Group) Corp. Ltd., Xi'an, 710069, P. R. China.

Email: yangdongyuan885@163.com; junwang.tang@ucl.ac.uk

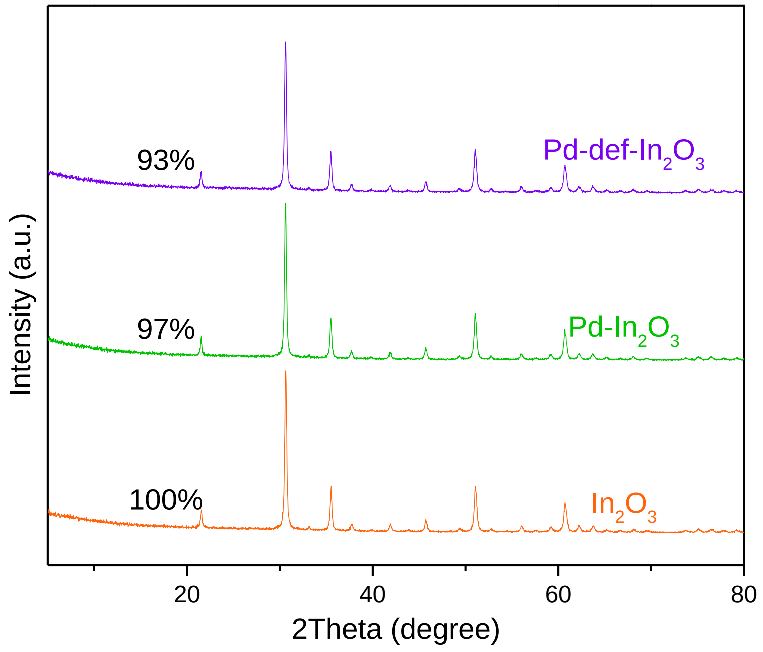

Figure S1. XRD patterns of In_2O_3, Pd-In_2O_3 and Pd-def-In_2O_3.

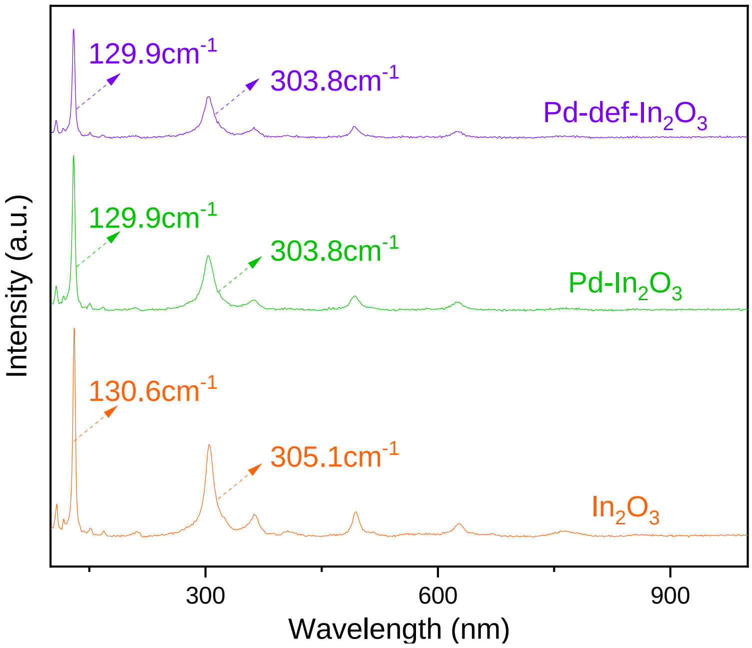

Figure S2. (a) Raman spectra of In_2O_3, Pd-In_2O_3 and Pd-def-In_2O_3.

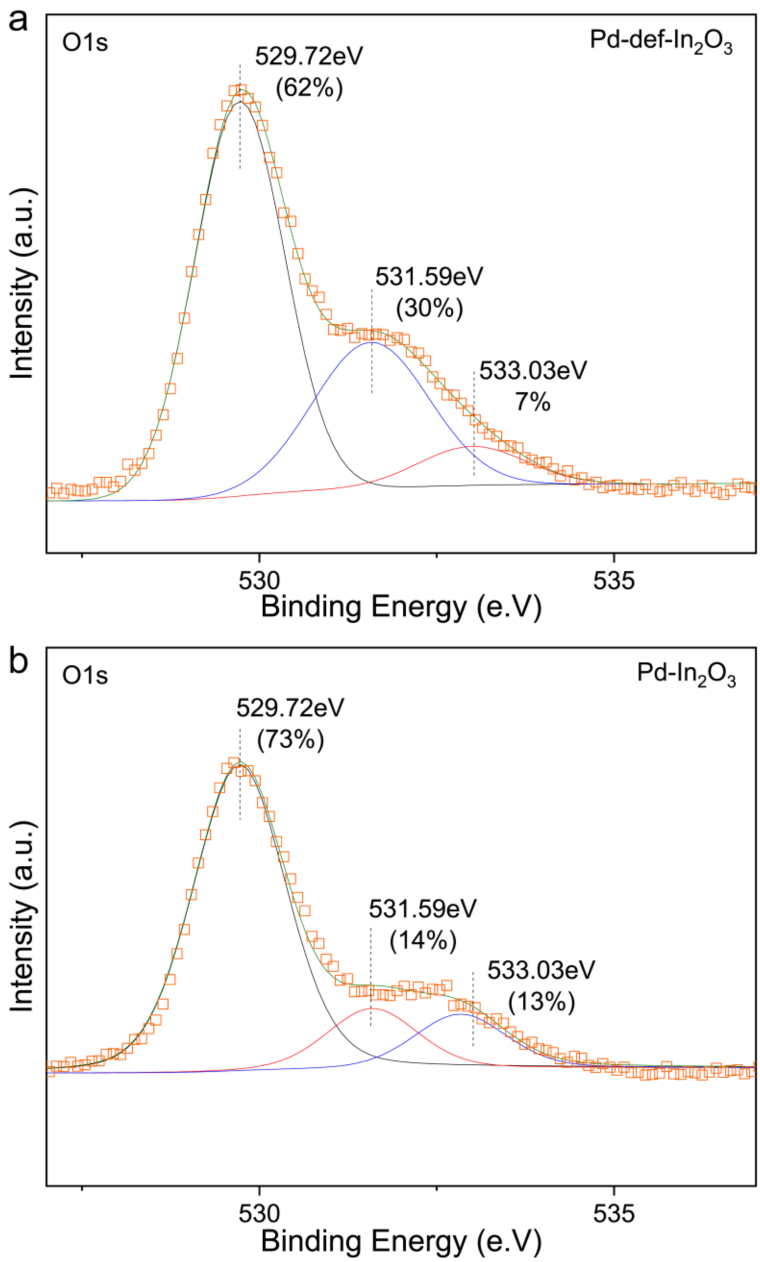

Figure S3. High-resolution of O_{1s} XPS spectra of (a) Pd-def-In_2O_3 and (b) Pd-In_2O_3.

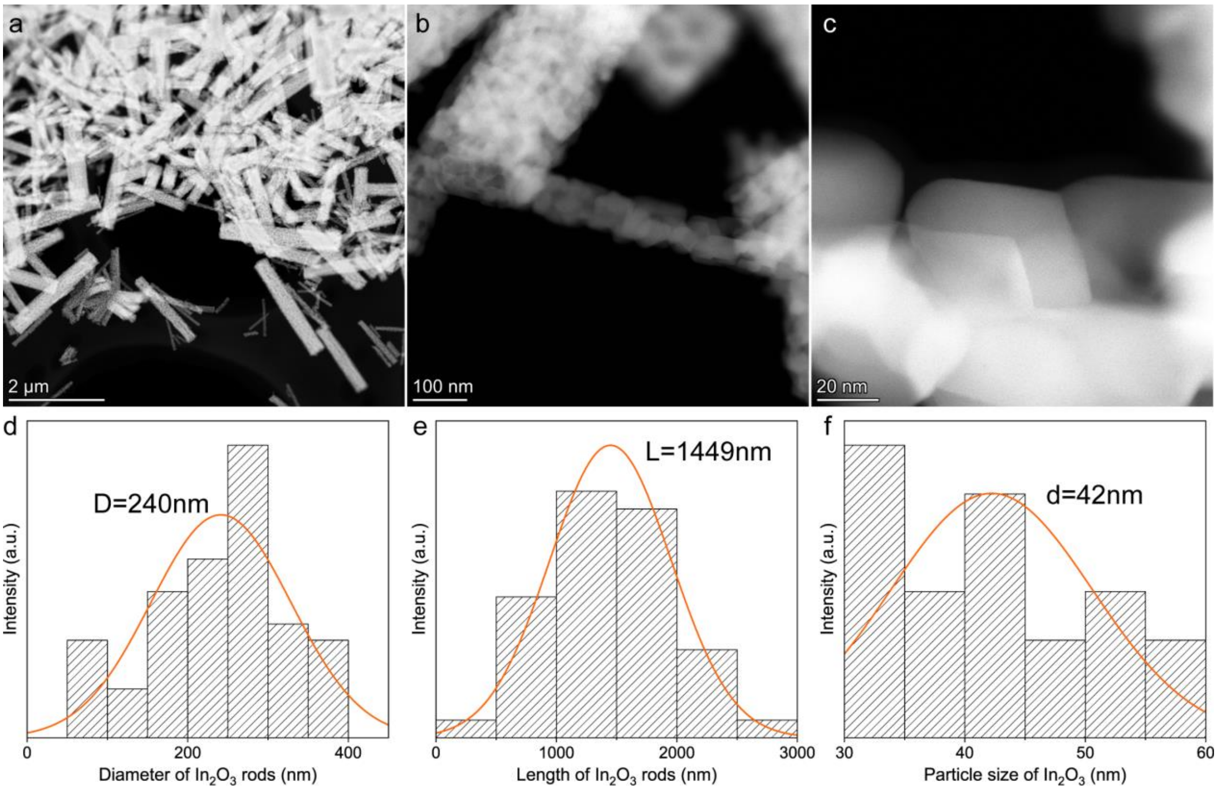

Figure S4. (a-c) HAADF-STEM images of Pd-def-In_2O_3. Size distribution of Pd-def-In_2O_3: (d) diameter and (e) length of the nanorods, (f) particle size of In_2O_3 crystalline.

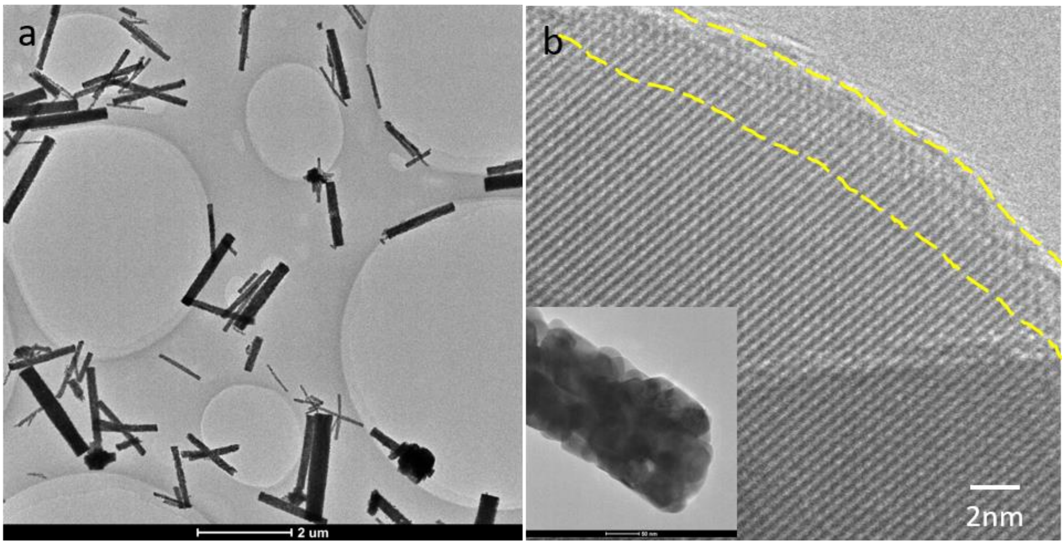

Figure S5. (a, b) HRTEM images of Pd-In_2O_3. In the region marked by yellow dash in panel b, no defective layer was observed on the edge of the Pd-In_2O_3 nanorods, suggesting the defect-lean structure of Pd-In_2O_3.

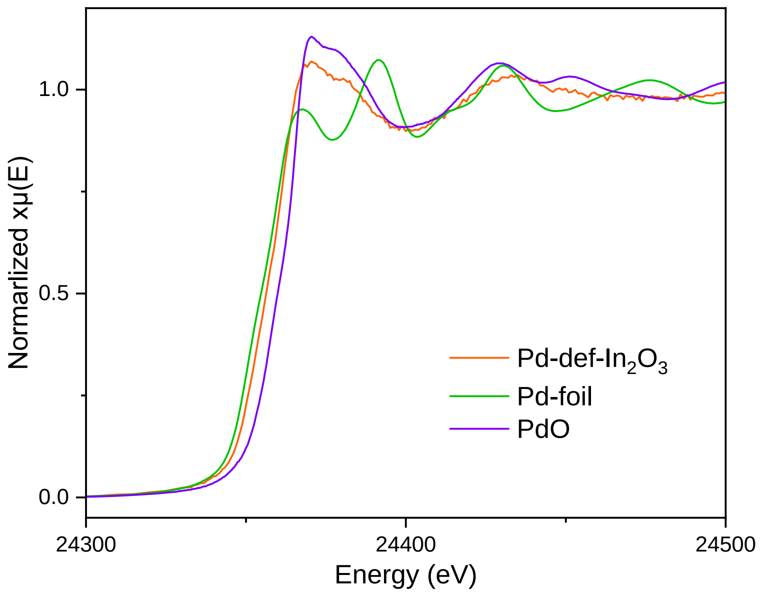

Figure S6. Normalized XANES spectra of the Pd K-edge of Pd-def-In_2O_3, PdO and Pd-foil.

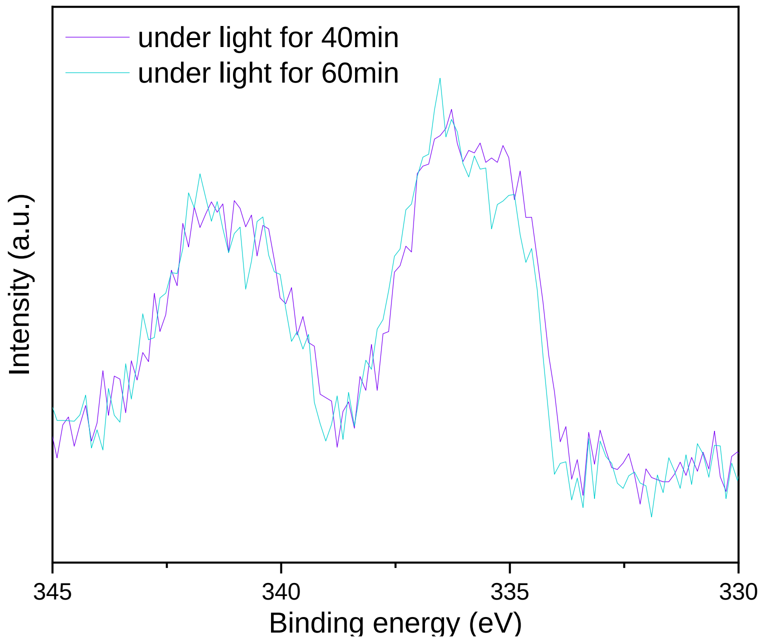

Figure S7. In-situ Pd_{3d} XPS spectra of $Pd_{0.3}$-def-In_2O_3 in dark and under light irradiation for 40 and 60min.

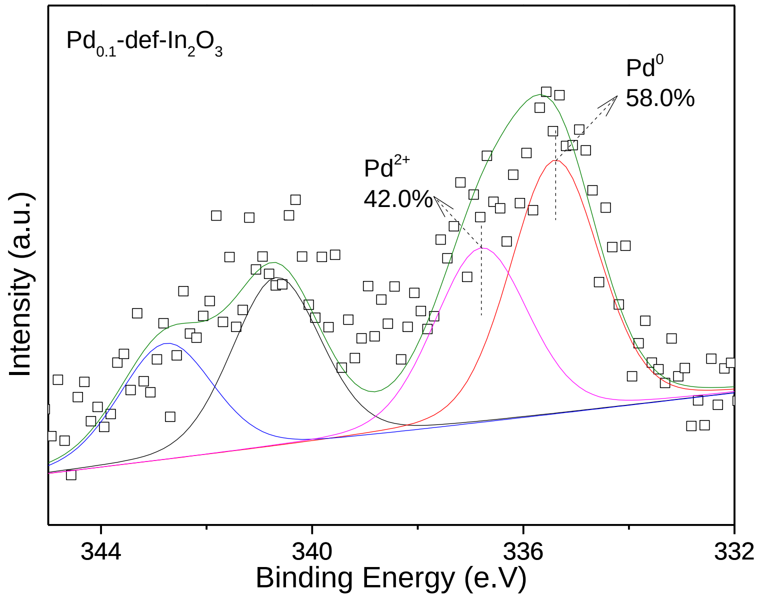

Figure S8. High-resolution Pd_{3d} XPS spectra of $Pd_{0.1}$-def-In_2O_3.

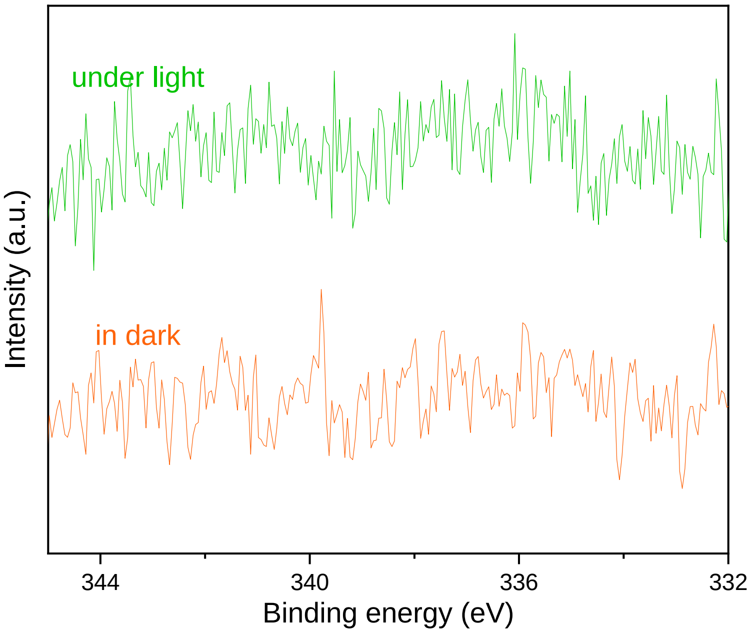

Figure S9. In-situ XPS spectra of $Pd_{0.1}$-def-In_2O_3 with extremely low Pd content in dark and under light irradiation.

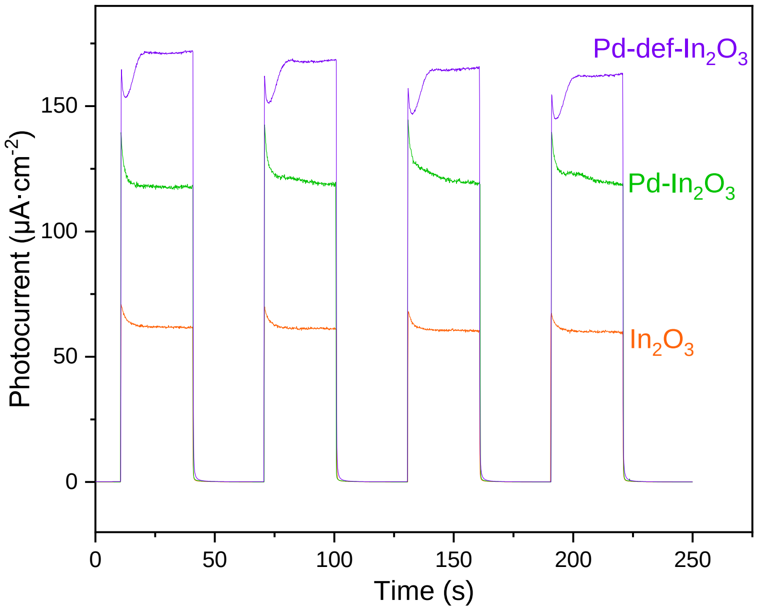

Figure S10. Photocurrent responses of In_2O_3, Pd-In_2O_3 and Pd-def-In_2O_3.

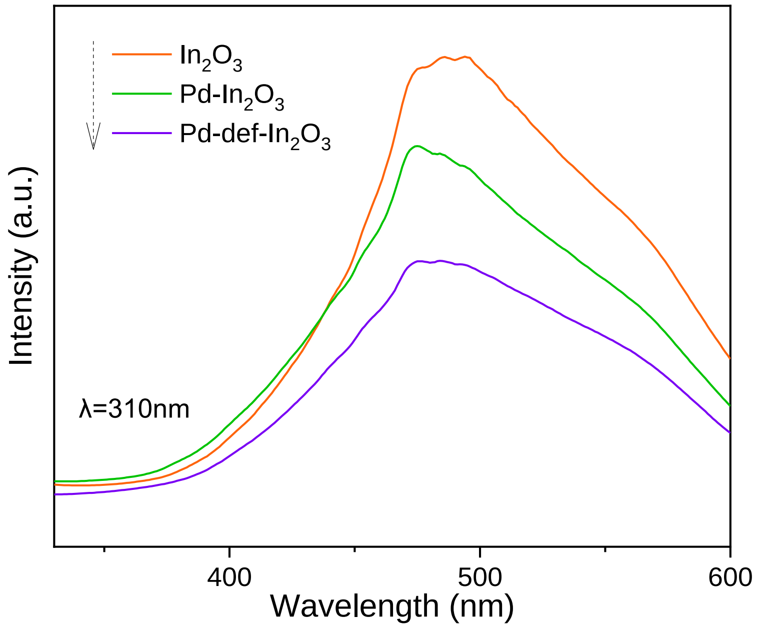

Figure S11. Steady-state PL spectra of In_2O_3, Pd-In_2O_3 and Pd-def-In_2O_3.

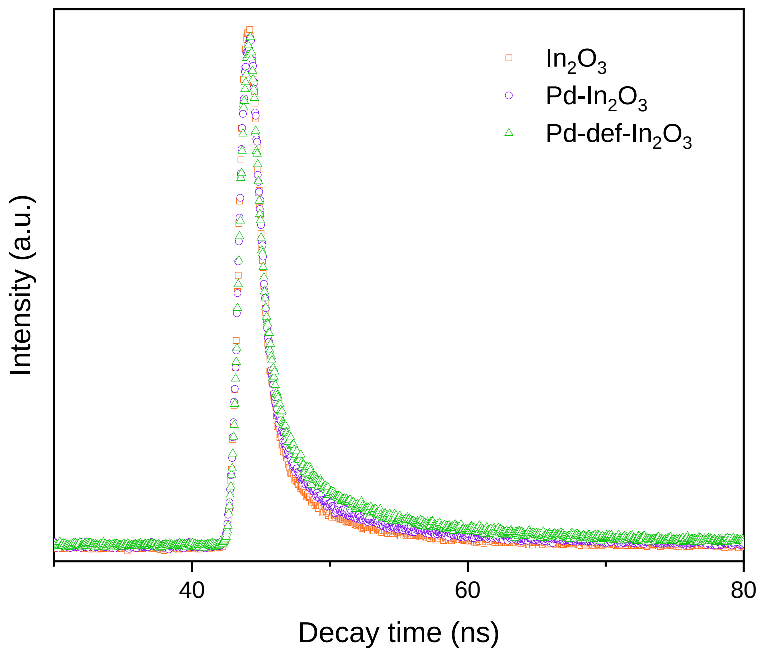

Figure S12. Time-decay PL spectra of In_2O_3, $Pd-In_2O_3$ and $Pd-def-In_2O_3$.

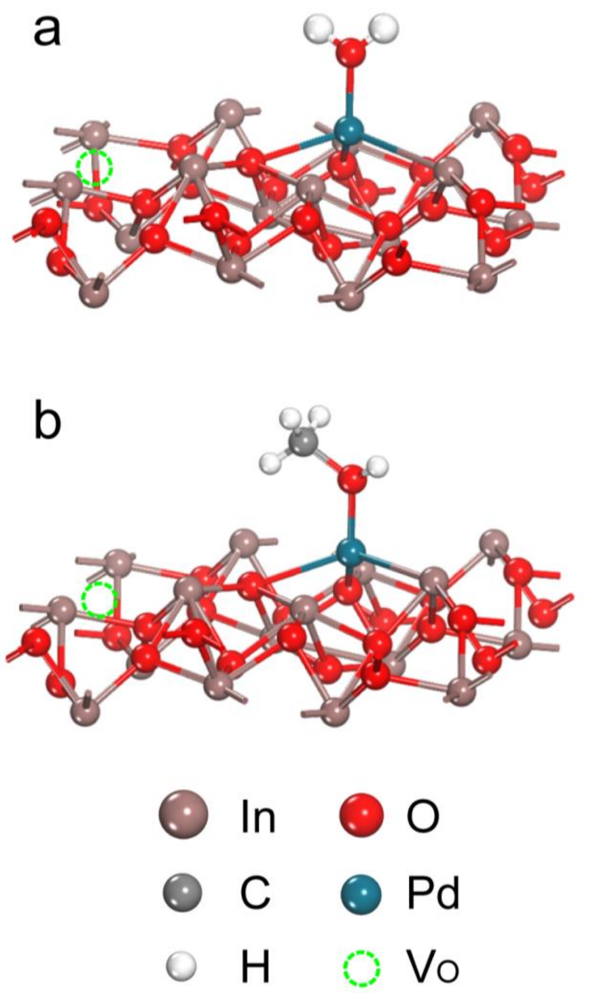

Figure S13. DFT calculation of optimized geometries of (a) H_2O and (b) CH_3OH on Pd-def-In_2O_3(far) with one oxygen vacancy far away from a Pd atom. The calculated adsorption energies were -1.76 and -1.40 eV, respectively.

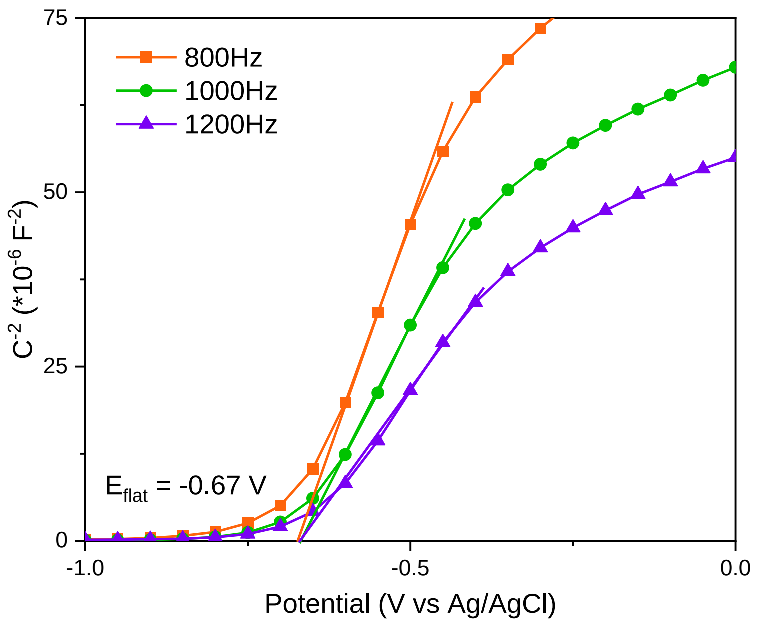

Figure S14. Mott-Schottky plots of In_2O_3 substrate with different frequencies.

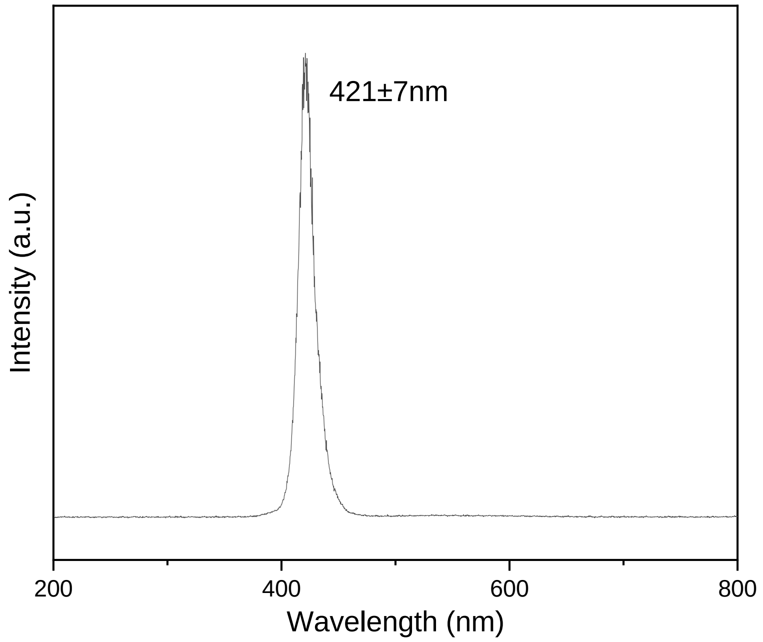

Figure S15. Spectrum of the 420 nm LED lamp.

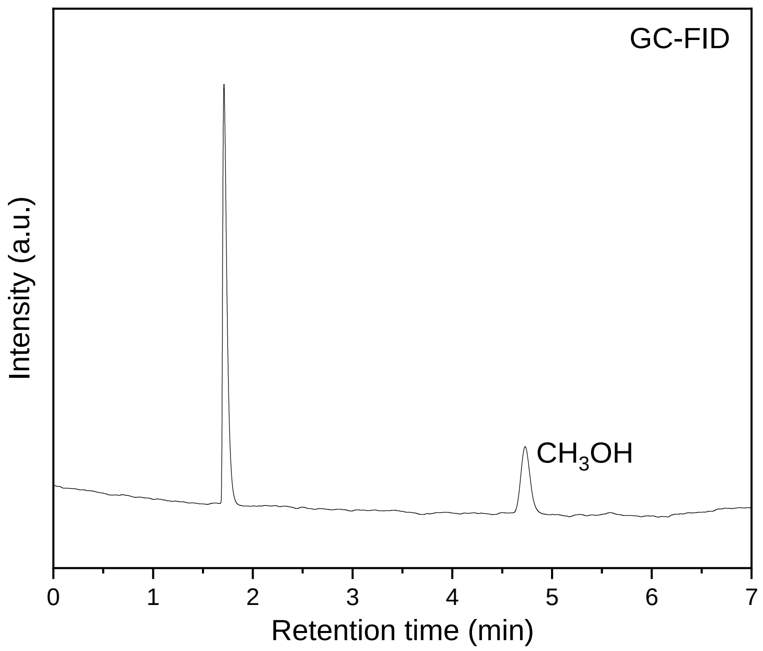

Figure S16. Representative curve for the detection of the liquid product by GC-FID method.

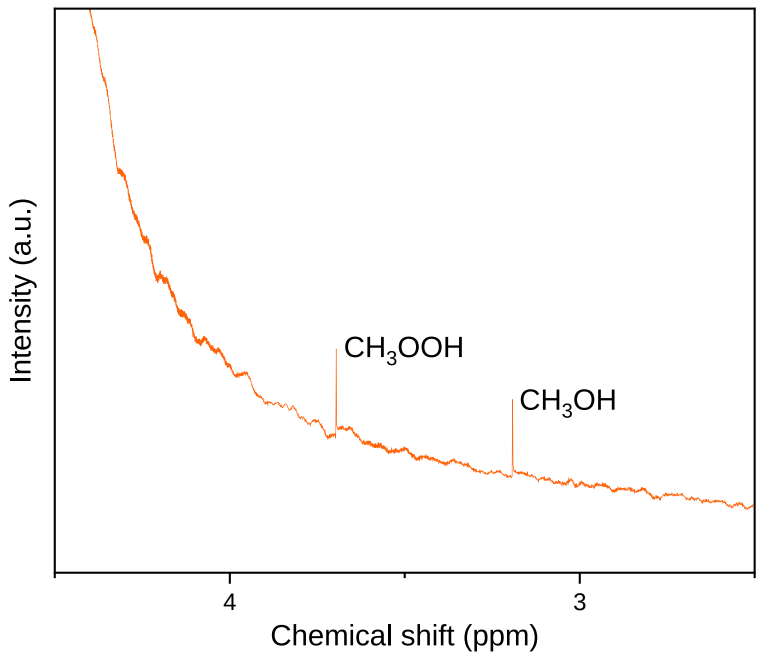

Figure S17. The representative ^{1}H NMR results to determine the molar ratio of CH_3OOH to CH_3OH.

Table S1. Contents of Pd, K and Cl elements in In_2O_3, Pd-In_2O_3 and Pd-def-In_2O_3 photocatalysts analysed by ICP-OES and IC methods.

	Pd (wt.%)	K (wt.%)	Cl (wt.%)
$Pd_{0.1}$-In_2O_3[a] (Pd-In_2O_3)	0.080	0.0082	0
$Pd_{0.01}$-def-In_2O_3	0.013	/	/
$Pd_{0.05}$-def-In_2O_3	0.049	/	/
$Pd_{0.1}$-def-In_2O_3 (Pd-def-In_2O_3)	0.083	0.0050	0
$Pd_{0.3}$-def-In_2O_3	0.284	/	/
$Pd_{0.5}$-def-In_2O_3	0.476	/	/

[a.] Pd-In_2O_3 was prepared with $(NH_4)_2PdCl_4$ as the Pd ions precursor, indicating 0.008 wt% K^+ measured by the ICP-OES method is the background nosie of the machine.

Table S2. Photocatalytic CH_4 conversion over different photocatalysts. Reaction conditions: 20 mg photocatalysts, 50 mL H_2O, 1 bar O_2, 19 bar CH_4, 25 °C and 3 h reaction time.

	CH_3OOH (μmol)	CH_3OH (μmol)	HCHO (μmol)	Total C1 (μmol)	CO_2 (μmol)
In_2O_3	0.0	0.0	0.95	0.95	0.0
Pt-In_2O_3	0.0	0.0	0.92	0.92	0.0
Pt-def-In_2O_3	0.0	0.0	1.02	1.02	0.0
Pd-In_2O_3	32.3	13.4	27.5	73.2	0.5
Pd-def-In_2O_3	107.6	37.9	34.3	179.7	1.1
Au-In_2O_3	0.0	0.0	1.4	1.4	0.0
Au-def-In_2O_3	0.0	0.0	2.7	2.7	0.0
$Pd_{0.01}$-def-In_2O_3	27.6	9.8	11.3	48.7	0.2
$Pd_{0.05}$-def-In_2O_3	47.6	24.2	19.0	90.8	0.4
$Pd_{0.3}$-def-In_2O_3	40.8	20.8	15.9	77.5	0.3
$Pd_{0.5}$-def-In_2O_3	15.4	6.3	4.2	26.0	0.3
In_2O_3/KCl[a]	0.0	0.0	0.97	0.97	0.0

[a] Photocatalytic CH_4 conversion over pristine In_2O_3 with the addition of KCl to the reaction solution. Molar ratio of KCl to Pd was two.

Table S3. Fitted PL lifetime from time-decay PL spectra of In_2O_3, Pd-In_2O_3 and Pd-def-In_2O_3.

Sample	α_1	τ_1 [ns]	α_2	τ_2 [ns]	τ [ns]
In_2O_3	0.98	49.9	6.21	50.1	3.60
Pd-In_2O_3	1.03	44.4	6.87	55.6	4.28
Pd-def-In_2O_3	1.00	36.8	7.32	63.2	4.99
